# A plasma fatty acid profile associated to type 2 diabetes development: from the CORDIOPREV study

**DOI:** 10.1007/s00394-021-02676-z

**Published:** 2021-10-05

**Authors:** Alejandro Villasanta-Gonzalez, Juan Francisco Alcala-Diaz, Cristina Vals-Delgado, Antonio Pablo Arenas, Magdalena P. Cardelo, Juan Luis Romero-Cabrera, Fernando Rodriguez-Cantalejo, Javier Delgado-Lista, Maria M. Malagon, Pablo Perez-Martinez, Matthias B. Schulze, Antonio Camargo, Jose Lopez-Miranda

**Affiliations:** 1 Lipids and Atherosclerosis Unit, Internal Medicine Unit, Reina Sofia University Hospital, Av. Menendez Pidal, s/n., 14004 Córdoba, Spain; 2 Department of Medicine, University of Cordoba, Córdoba, Spain; 3 Instituto Maimonides de Investigación Biomedica de Cordoba (IMIBIC), Córdoba, Spain; 4 CIBER Fisiopatología de la Obesidad y Nutrición (CIBEROBN), Instituto de Salud Carlos III, Madrid, Spain; 5 Biochemical Laboratory, Reina Sofia University Hospital, Córdoba, Spain; 6 Department of Cell Biology, Physiology and Immunology, University of Cordoba, Córdoba, Spain; 7 German Center for Diabetes Research (DZD), München-Neuherberg, Germany; 8 Department of Molecular Epidemiology, German Institute of Human Nutrition Potsdam-Rehbrücke, Nuthetal, Germany; 9 Institute of Nutrition Science, University of Potsdam, Nuthetal, Germany

**Keywords:** Type 2 diabetes, Disease prediction, Fatty acids, COX, FA Score

## Abstract

**Purpose:**

The prevalence of type 2 diabetes mellitus (T2DM) is increasing worldwide. For this reason, it is essential to identify biomarkers for the early detection of T2DM risk and/or for a better prognosis of T2DM. We aimed to identify a plasma fatty acid (FA) profile associated with T2DM development.

**Methods:**

We included 462 coronary heart disease patients from the CORDIOPREV study without T2DM at baseline. Of these, 107 patients developed T2DM according to the American Diabetes Association (ADA) diagnosis criteria after a median follow-up of 60 months. We performed a random classification of patients in a training set, used to build a FA Score, and a Validation set, in which we tested the FA Score.

**Results:**

FA selection with the highest prediction power was performed by random survival forest in the Training set, which yielded 4 out of the 24 FA: myristic, petroselinic, α-linolenic and arachidonic acids. We built a FA Score with the selected FA and observed that patients with a higher score presented a greater risk of T2DM development, with an HR of 3.15 (95% CI 2.04–3.37) in the Training set, and an HR of 2.14 (95% CI 1.50–2.84) in the Validation set, per standard deviation (SD) increase. Moreover, patients with a higher FA Score presented lower insulin sensitivity and higher hepatic insulin resistance (*p* < 0.05).

**Conclusion:**

Our results suggest that a detrimental FA plasma profile precedes the development of T2DM in patients with coronary heart disease, and that this FA profile can, therefore, be used as a predictive biomarker.

**Clinical Trials.gov.Identifier:**

NCT00924937.

**Supplementary Information:**

The online version contains supplementary material available at 10.1007/s00394-021-02676-z.

## Introduction

The prevalence of type 2 diabetes mellitus (T2DM) has increased worldwide over the last few years and is expected to grow substantially over the next few decades. Characterized by elevated blood glucose levels, the etiology of T2DM is complex, and insulin resistance (IR) and impaired beta-cell function are critical determinants of this disease [1]. Lifestyle factors, including physical inactivity and unhealthy diets, are important factors contributing to T2DM development [2]. Patients with T2DM are at higher risk of cardiovascular disease (CVD), a class of diseases that involve the heart or blood vessels, and most deaths of diabetic patients are due to this pathology [3]. In this context, the simultaneous presence of both CVD and T2DM considerably increases the risk of macrovascular complications and mortality [4]. It is, therefore, especially important to devise strategies to prevent the development of T2DM in patients with CVD.

Lifestyle modifications, such as physical exercise and a healthy diet, may delay the appearance of T2DM or even prevent the development of this disease [5]. In this context, a low-fat (LF) diet or a Mediterranean (MED) diet have been put forward as reasonable ways to delay T2DM development [6, 7]: the MED diet, rich in olive oil, seems to provide cardiovascular benefits and increase insulin sensitivity [7] and the LF diet contains < 30% energy from total fat and is recommended by the American Diabetes Association (ADA) [8].

The importance of nutrition in the management and prevention of T2DM through its effect on weight and metabolic control is clear [9]. However, nutritional strategies are not fully effective and/or their effectiveness is dependent on the individual. In short, despite dietary recommendations, there is no globally supported diet to prevent diabetes, and population-specific and individual factors may determine success in preventing T2DM using different nutritional strategies [5]. Therefore, the identification of biomarkers for early detection of T2DM risk and/or better prognosis of T2DM and to assess the efficacy of targeted interventions is essential.

In the last few years, several studies have shown that plasma lipid species are a determinant factor for T2DM development, suggesting that changes in this profile may trigger the development of this disease. For example, a plasma lipidomic profile has been described for T2DM prediction in a high cardiovascular risk population [10], and has also been described for evaluating the cardiovascular risk [11]. Moreover, the specific fatty acids (FA) composition in plasma phospholipids has also been shown to be useful for assessing T2DM risk [12]. Here, several works have studied the association between FA and the probability of diabetes development. However, most of these studies analyzed individual FA or FA groups, such as saturated FA [13, 14] or polyunsaturated omega 3 or 6 [15–17], rather than analyzing the full FA profile. In fact, FA are not merely a source of energy, but play vital roles in metabolic homeostasis, serving as precursors of signaling molecules, and are basic components involved in the architecture and function of cellular membranes and functional lipids, binding to various receptors and transcription factors [18, 19].

Based on this previous evidence, we hypothesized that a specific plasma FA profile rather individual fatty acids may precede T2DM development in coronary heart patients, and, therefore, could be used as a biomarker to assess T2DM risk. We aimed to evaluate the plasma FA profile as an associated biomarker for T2DM development in the non-T2DM coronary heart disease patients from the CORDIOPREV study.

## Material and methods

### Study subjects

The current work was conducted within the framework of the Coronary Diet Intervention with Olive Oil and Cardiovascular Prevention study (CORDIOPREV; Clinical Trials.gov.Identifier: NCT00924937), an ongoing prospective, randomized, open, controlled trial of 1002 patients with coronary heart disease (CHD), which develops when the coronary arteries become too narrow by atherosclerotic processes, who were receiving conventional treatment and had their last coronary event over 6 months before enrollment in one of two different dietary models (a Mediterranean (MED) diet and a low-fat (LF) diet) over a period of 7 years [20]. Treatment of CHD highly depends on any patient’s characteristics. Lifestyle management, with a healthy diet, exercise and tobacco avoiding is crucial. Main family drugs usually used include: cholesterol-modifying medications, antiagregants, beta-blockers, calcium channel blockers, Angiotensin-converting enzyme (ACE) inhibitors and angiotensin II receptor blockers (ARBs), and Nitroglycerin as a rescue drug.

The patients were recruited between November 2009 and February 2012, mostly at the Reina Sofia University Hospital (Cordoba, Spain), but other health centers from the Cordoba and Jaen provinces were also included. The eligibility criteria, design and methods of the CORDIOPREV clinical trial have been reported elsewhere [20]. In summary, patients were eligible if they were aged between 20 and 75 years, had established CHD without clinical events in the last 6 months, were asked to follow a long-term dietary intervention and did not have severe diseases or an estimated life expectancy of less than 5 years. All patients gave their written informed consent to participate in the study. The trial protocol and all amendments were approved by the local ethic committees, following the Helsinki declaration and good clinical practices. The experimental protocol conformed to international ethical standards.

We included patients (462 out of 1002) who had not been diagnosed with T2DM at the beginning of the CORDIOPREV study, according to the American Diabetes Association (ADA) T2DM diagnosis criteria [21]. From a total of 462 patients who did not have a T2DM diagnosis at the beginning of the study, 3 patients were excluded from the study because we did not have their plasma fatty acid data due to technical difficulties in the analytical procedure. Furthermore, of these 459 patients, who had not been clinically diagnosed with T2DM at the study baseline, 107 patients developed T2DM, according to the ADA diagnosis criteria, after a median follow-up of 60 months. The incidence of T2DM was assessed every year according to the ADA T2DM criteria: fasting plasma glucose ≥ 126 mg/dL or 2 h plasma glucose in the 75 g OGTT ≥ 200 mg/dL or HbA1c levels ≥ 6.5%. The baseline characteristics of the subjects in the study are shown in Supplementary Table 1.

### Study design

The study design has been previously described [20, 22]. Briefly, participants were randomized to receive two diets: a MED diet or an LF diet. The LF diet consisted of < 30% total fat (< 10% saturated fat, 1214% monounsaturated fatty acids (MUFA) fat, and 6–8% polyunsaturated fatty acids (PUFA) fat), 15% protein, and a minimum of 55% carbohydrates. The MED diet comprised a minimum 35% of calories as fat (22% MUFA fat, 6% PUFA fat, and < 10% saturated fat), 15% proteins, and a maximum of 50% carbohydrates. In both diets, the cholesterol content was adjusted to < 300 mg/d.

### Dietary assessment

At the beginning of the study and every year, each patient had a face-to-face interview with a nutritionist to fill in a 137-item semi-quantitative food frequency questionnaire, validated for Spain [23], and well as a validated 14-item questionnaire of adherence to the Mediterranean diet to produce a Mediterranean diet score [24]. MED and LF diets were designed to provide a wide variety of foods, including vegetables, fruit, cereals, potatoes, legumes, dairy products, meat and fish. Participants in both intervention groups received the same intensive dietary counseling. Nutritionists administered personalized individual interviews at inclusion and every 6 months, and quarterly group education sessions were held with up to 20 participants per session and separate sessions for each group.

### Clinical plasma parameters

At the beginning of the study and every year, venous blood was collected in tubes containing EDTA and used to analyze the participants’ biochemical variables. Lipid variables, serum insulin and plasma glucose were determined, as previously reported [25].

### Oral glucose tolerance test (OGTT)

Before starting the test, the patients had fasted (food/drugs) for 12 h and were asked to refrain from smoking during the fasting period and from alcohol intake during the preceding 7 days. They were also asked to avoid strenuous physical activity the day before the test was given. At 8:00 a.m., the patients were admitted to the laboratory and an OGTT (75 g dextrose monohydrate in 250 mL water) was performed with 0, 30, 60, 90, and 120 min sampling to establish plasma glucose and insulin levels. OGGT were performed during the 5 years of follow-up. The OGTT-derived indexes were calculated as previously described [25]. Briefly, the Insulin Sensitivity Index (ISI = 10.000÷√([fasting plasma glucose × fasting plasma insulin] × [mean glucose in OGTT × mean insulin in OGTT]), Disposition Index (DI = ISI × [AUC30 min insulin/AUC30 min glucose], where AUC30 min is the area under the curve between baseline and 30 min of the OGTT for insulin (pmol/L) and glucose (mmol/L) measurements, respectively, calculated by the trapezoidal method), Hepatic Insulin Resistance Index (HIRI = (fasting plasma insulin × fasting plasma glucose), Homeostatic Model Assessment Insulin Resistance (HOMA-IR = [fasting plasma insulin (mU/L) × 20)/(fasting plasma glucose (mmol/L) − 3,5)] and Insulinogenic Index (IGI = [plasma insulin at 30 min − fasting plasma insulin (mU/L))/(plasma glucose at 30 min − fasting plasma glucose(mg/dL)].

### GC–MS analysis of plasma fatty acid

Chromatographic separation was carried out by a gas chromatograph (Agilent 7890B) coupled to a mass spectrometer (Agilent 5977A), using a SPTM 2560 fused silica column (100 m × 0.25 mm, 0.25 μm film thickness) from Supelco (Bellefonte, PA, USA). The gas chromatography-mass spectrometry (GC–MS) analysis was performed according to the following conditions: injector temperature, 250 °C; injection in splitless mode; gas flow, 0.6 mL min^–1^ and injection volume, 1 μL. The oven temperature was programmed as follows: initial temperature 100 °C, hold for 5 min; ramp at 4 °C min^–1^ up to 240 °C, hold for 20 min. The total analysis time was 60 min, with 4 additional min necessary for re-establishing the initial conditions. The single quadrupole mass spectrometer was operated in the full scan mode, with the instrumental temperatures set at 250, 250, and 180 °C for transfer line, source, and quadrupole, respectively. The electron energy was set at 70 eV, and the data acquisition was carried out in an *m*/*z* range from 45 to 750 *m*/*z* and with a solvent delay for 14.5 min.

### Identification and confirmatory analysis of FA

Qualitative Analysis software (version 7.0, Agilent Technologies, Santa Clara, CA, USA) was used to process the data obtained by GC–MS. Treatment of raw data files started with the deconvolution of potential molecular features by the algorithm included in the software, which considered all ions exceeding 3000 counts for the absolute area parameter. The NIST Mass Spectral Search Program v.11.0 (NIST, Washington, DC, USA) was used for the spectral search (Mainlib and Replib libraries). Tentative identification was reported when the correlation between experimental and database spectra was above 0.85 in normal search mode. Confirmatory analysis was carried out by analysis of a FAMEs multistandard from Sigma-Aldrich (Supelco 37 component FAME mix).

### Statistical analysis

RStudio and SPSS statistical software (IBM SPSS Statistics version 21.0) were used for the statistical analysis of data. The normal distribution of variables was assessed using the Kolmogorov–Smirnov test. Data are represented as the mean ± SEM for continuous variables and as frequencies for categorical variables. *P* values ≤ 0.05 were considered statistically significant. The statistical differences in the metabolic variables between groups were evaluated by One-way ANOVA. Qualitative variables were compared using the Chi-square test. A repeated-measures ANOVA test was used to determine the statistical differences between indexes during the OGTT at baseline and after 5 years of follow-up. The post hoc statistical analysis was completed using Bonferroni's multiple comparison tests.

### COX analysis

We performed a Cox proportional hazards regression analysis with each one of 24 FA detected. Baseline individual FA values were normalized by log-transformation and scaled in multiples of 1 standard deviation (SD). In the Cox proportional hazards regression analysis, we looked for independent variables which were related to variations in the risk function of some patients with respect to a specific event studied. Therefore, the Cox regression looked for the relationship between the risk of T2DM development, and the plasma fatty acids levels. Moreover, a Cox regression yields a hazard ratio, that indicates which patients have a lower or higher risk of suffering the event according to the variable or variables included in the analysis.

In addition, we performed a random classification of patients into two different datasets: the Training set, with 275 patients (60% of the total) and the Validation set, with 184 patients (40% of the total). To analyze the association of FA with T2DM development, we calculated the Hazard Ratio (HR) of each FA in the Training set, for each SD increase in baseline individual FA levels.

### Random survival forest

To select FA with a higher predictive power for T2DM, we made a preselection in the Training set by applying random survival forest (RSF), in combination with a backward selection procedure in the training set [26]. RSF utilizes a collection of decision trees for prediction and sorts the different variables by their importance according to the time of the event, in this case the development of T2DM since the beginning of the study. Therefore, RSF is a very useful tool to reduce the number of metabolites in prospective cohorts, selecting those metabolites with greater importance related to the event of interest of the study. Consequently, we can eliminate the fatty acids which do not contribute to the T2DM development, and select a model with the lowest prediction error, which includes the fatty acids with higher predictive power.

Meanwhile, we validated the results obtained in the training set in the group of patients included for such end in the validation set. We used the same random classification of patients in the two different datasets: training and validation sets, and the baseline individual FA values normalized by log-transformation and scaled in multiples of 1 SD.

### Random survival forest-based FA Score building

We performed a Cox proportional hazards regression analysis with FA selected in the Training set to determine the jointly potential use of these FA as an independent predictor of T2DM development. Further, to assess the relationship between plasma FA profile and T2DM development, we built a Fatty Acid Score (FA Score) for each patient by multiplying the coefficients of a FA, obtained in the previous COX analysis performed in the Training set, by its respective value and then adding the products obtained for a certain patient. Moreover, we created the Score in the Training and Validation sets, for the purpose of validate the selection and the coefficients obtained in the two previous steps, which were only performed in the Training set. Furthermore, we classified patients according to the Score generated into median, tertiles and quartiles, and performed the COX proportional hazards regression with each one of those variables. We classified the same population with different cut-off points to evaluate the association of the generated FA Score.

## Results

### Baseline characteristics of the participants

The baseline characteristics of the subjects in the study are shown in Supplementary Table 1. We observed that the values of body mass index (BMI), weight, waist circumference, serum triacylglycerols (TAG), HbA1c, fasting glucose, fasting insulin and HOMA-IR were higher, and the ISI, IGI and DI values were lower in the Incident-DIAB group compared with the Non-DIAB group (all, *p* < *0.05*).

### Individual FA plasma levels as a predictor of T2DM development

We performed a Cox proportional hazards regression analysis to determine the potential association of individual fatty acids (FA) and type 2 diabetes (T2DM) development. To do this, we calculated the Hazard Ratio (HR) for each standard deviation (SD) increase in baseline individual FA levels in the Training set. Once performed, the analysis was adjusted by age, gender, diet consumed, BMI, treatment with statins, HDL-c and TAG plasma levels, on the basis that these variables are considered risk factors for T2DM development and statins are diabetogenic, in addition to the adjustment for diet as patients consumed a LF diet or the Med diet. (Supplementary Table 2).

From the Cox proportional hazards regression analysis of 24 FA detected, 2 of them showed a statistically significant association with T2DM development, α-linolenic acid (ALA) (18:3n-3) and arachidonic acid (AA) (20:4n-6). Regarding ALA, we observed an unadjusted HR of 0.74 (95% CI 0.57–0.96) and an adjusted HR of 0.73 (95% CI 0.55–0.97), and for AA an unadjusted HR of 1.23 (95% CI 0.95–1.61) and an adjusted HR of 1.35 (95% CI 1.02–1.81), for each SD increase.

In addition, patients were categorized in ascending tertiles by levels of these fatty acids, ALA and AA (Supplementary Fig. 1), in the Training and Validation set. When we categorized our population according to ALA levels we observed a lower risk in patients with Intermediate (unadjusted HR: 0.48; 95% CI 0.26–0.88, and adjusted HR: 0.42; 95% CI 0.22–0.82), and Higher (unadjusted HR: 0.55; 95% CI 0.30–1.00, and adjusted HR: 0.56; 95% CI 0.30–1.07) values of ALA in the Training set, whereas in the Validation set, we obtained similar results, but not significant in the patients with Intermediate (unadjusted HR: 0.52; 95% CI 0.26–1.06, and adjusted HR: 0.62; 95% CI 0.30–1.31) and Higher (unadjusted HR: 0.56; 95% CI 0.28–1.14, and adjusted HR: 0.61; 95% CI 0.29–1.29) levels of ALA, taken subjects with lower ALA levels as reference.

On the other hand, when patients were classified by AA concentration, we did not obtain significant results either in Training or Validation. In the Training set, we had a higher HR in patients with Intermediate (unadjusted HR: 1.72; 95% CI 0.91–3.27, and adjusted HR: 1.91; 95% CI 0.98–3.74) and Higher (unadjusted HR: 1.52; 95% CI 0.77–2.92, and adjusted HR: 1.74; 95% CI 0.85–3.55) levels of AA, while in Validation Set, we observed a slightly higher HR in patients with Intermediate (unadjusted HR: 1.46; 95% CI 0.72–2.96, and adjusted HR: 1.46; 95% CI 0.69–3.08), and no differences in subjects with Higher (unadjusted HR: 0.96; 95% CI 0.44–2.06, and adjusted HR: 1.02; 95% CI 0.44–2.34) values of AA, compared with patients with lower AA concentration.

### Random survival forest

**Fig. 1:**
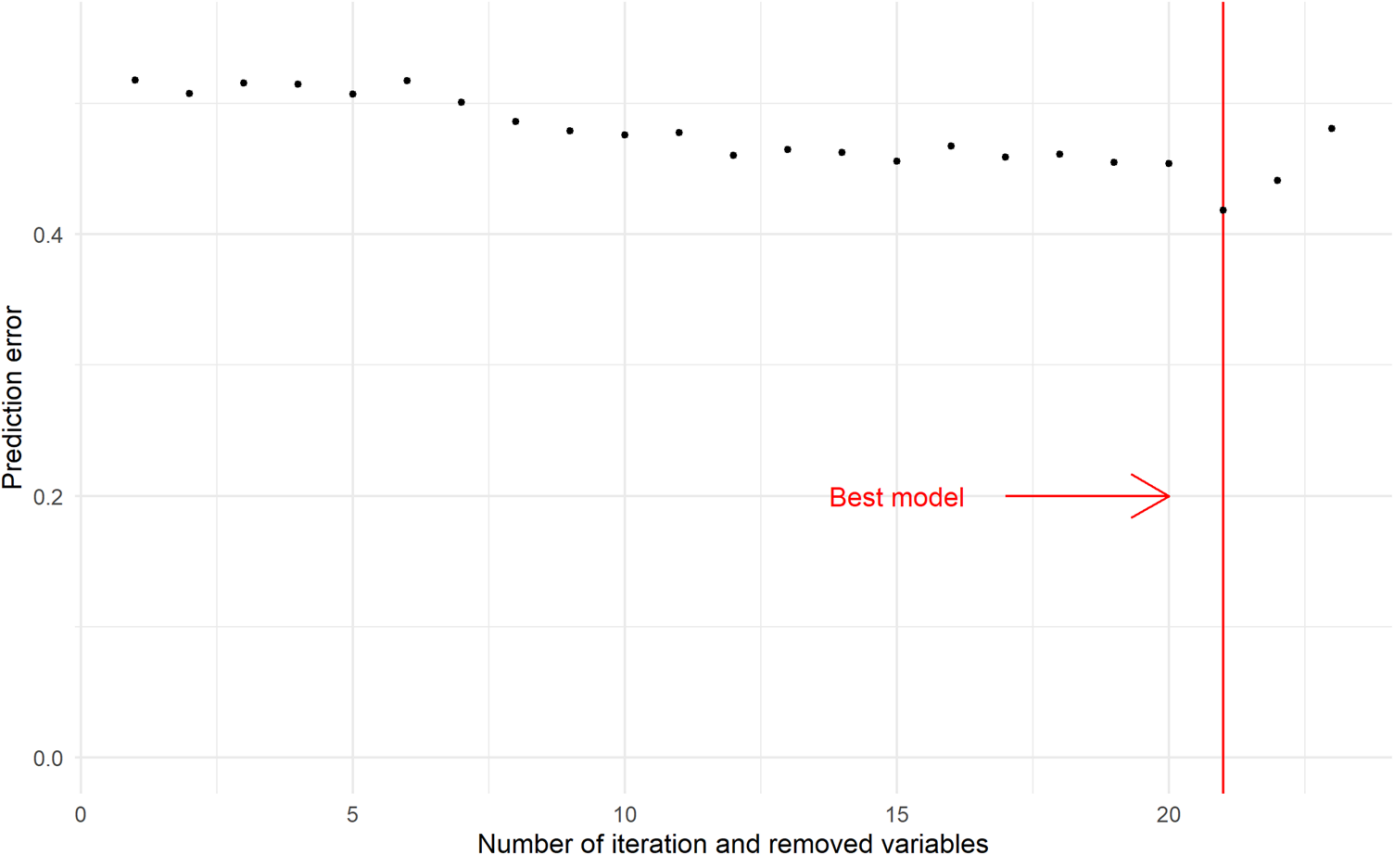
Selection of the best model by Random Survival Forest (RSF). Selection in the Training set of fatty acids with a higher predictive power for type 2 diabetes, by applying an RSF in combination with a backward selection procedure

We performed a random survival forest (RSF) in combination with a backward selection procedure in the Training set to select the FA with a higher predictive power for T2DM development and a minimum prediction error (Fig. 1). From a total of 24 fatty acids, 4 fatty acids were selected by RSF and included in the model with a lower prediction error: myristic acid (MA) (14:0), petroselinic acid (PA) (18:1n-12), α-linolenic acid (ALA) (18:3n-3) and arachidonic acid (AA) (20:4n-6).

### Association between RSF-selected fatty acids and T2DM development by COX regression analysis

Furthermore, to establish the relationship of the selected fatty acids jointly with the development of diabetes, Cox proportional hazards regression analysis was performed with these fatty acids together in the Training set (Table 1). Once performed, the analysis was adjusted by age, gender, diet, BMI, treatment with statins, HDL-c and TAG plasma levels, on the basis that these variables are considered risk factors for T2DM development and statins are diabetogenic, in addition to the adjustment for diet as patients consumed a LF diet or the Med diet. The HR obtained for these FA, per SD increase, were as follows: for MA, we found an unadjusted HR of 1.09 (95% CI 0.82–1.46) and an adjusted HR of 1.08 (95% CI 0.77–1.52); for PA an unadjusted HR of 0.93 (95% CI 0.74–1.15) and an adjusted HR of 0.97 (95% CI 0.70–1.35); for ALA, we obtained an unadjusted HR of 0.76 (95% CI 0.59–0.99) and an adjusted HR of 0.76 (95% CI 0.57–1.01); and finally, for AA we found an unadjusted HR of 1.24 (95% CI 0.91–1.68) and an adjusted HR of 1.34 (95% CI 0.97–1.88). In summary, when we analyzed the four FA selected together, we observed that two were directly associated with T2DM development: MA and ARA; meanwhile, PA and ALA were associated with a lower risk of T2DM, with ALA the fatty acid with the highest weight in the model.

**Table 1:** Association between fatty acids selected in the RSF and T2DM development, per SD increase

		Coeff	HR		95% CI for HR				Linear trend	
					Lower		Upper			
Model 1										
Myristic acid C14:0	0.087			1.09		0.82		1.46		0.559
Petroselinic acid C18:1n-12	− 0.077			0.93		0.74		1.15		0.492
α-Linolenic acid C18:3n-3	− 0.269			0.76		0.59		0.99		0.047*
Arachidonic acid C20:4n-6	0.211			1.24		0.91		1.68		0.177
Model 2										
Myristic acid C14:0	0.079			1.08		0.77		1.52		0.649
Petroselinic acid C18:1n-12	− 0.029			0.97		0.70		1.35		0.860
α-Linolenic acid C18:3n-3	− 0.278			0.76		0.57		1.01		0.060º
Arachidonic acid C20:4n-6	0.299			1.34		0.97		1.88		0.078º

Model 1 was unadjusted, and Model 2 was adjusted by age, gender, diet, BMI, treatment with statins, HDL-c and TAG plasma levels

*Statistically significant

ºTrend to statistical significance

### A FA profile-based score associated with T2DM development

We built a Fatty Acid Score (FA Score) to assess the relationship between the FA profile associated with T2DM (RSF-selected fatty acid) and the development of this disease (see Materials and Methods). We next performed a COX proportional hazards regression analysis, and once performed, adjusted it by age, gender, diet, BMI, treatment with statins, HDL-c and TAG plasma levels, to test the T2DM risk according to the FA Score generated. We found an unadjusted HR of 2.72 (95% CI 2.14–3.45) and an adjusted HR of 3.15 (95% CI 2.04–3.37) in the Training set, while in the Validation set we obtained an unadjusted HR of 2.30 (95% CI 1.62–3.20) and an adjusted HR of 2.14 (95% CI 1.50–2.84), per standard deviation unit.

Furthermore, we classified patients according to the score generated by ascending tertiles, quartiles and median and evaluated the association between the FA Score and T2D development by COX proportional hazards regression, in the Training and in the Validation set, separately. The different cutoff points were done for the purpose of evaluating the association between the development of T2DM and the generated FA Score.

**Fig. 2:**
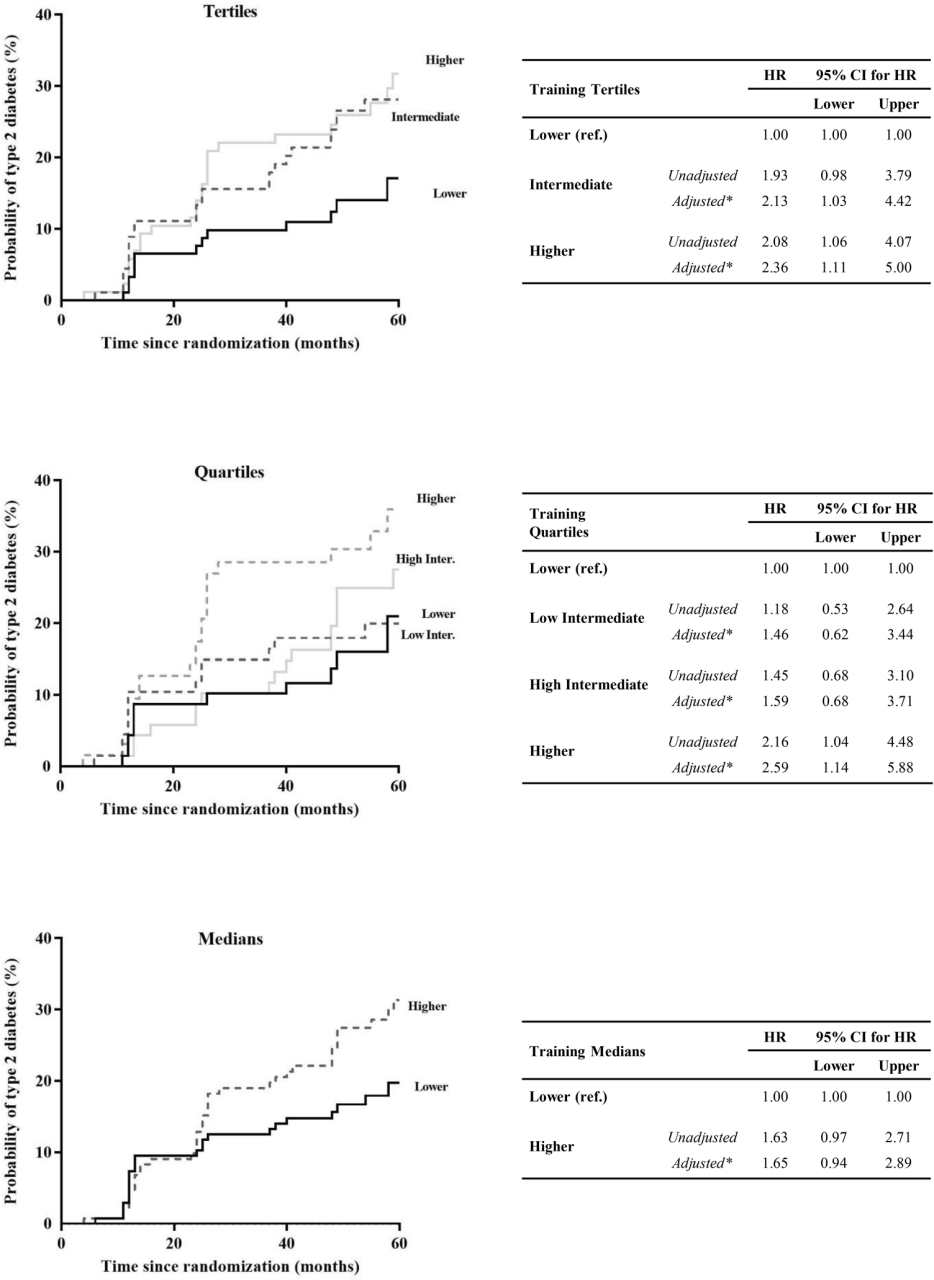
Disease-free survival by COX proportional hazards regression analysis according to FA Score in the Training set. Patients from the Training set were categorized according to the FA Score by tertiles, quartiles and median (ascending order). *This model was adjusted for age, gender, BMI, diet, treatment with statins, HDL-c and TG plasma levels. The hazard ratio (HR) between groups was calculated

In the Training set (Fig. 2), when we classified the patients by tertiles of the FA Score, we observed that patients classified in the Intermediate (unadjusted HR: 1.93; 95% CI 0.98–3.79, and adjusted HR: 2.13; 95% CI 1.03–4.42,) and Higher (unadjusted HR: 2.08; 95% CI 1.06–4.07, and adjusted HR: 2.36; 95% CI 1.11–5.00) FA Score groups, showed a greater risk of T2DM development than patients categorized with a Lower FA Score group, which was the reference group. The second classification by quartiles of the FA Score yielded consistent results, showing that the patients classified in the Higher FA Score (unadjusted HR: 2.16; 95% CI 1.04–4.48, and adjusted HR: 2.59; 95% CI 1.14–5.88) had a higher risk of diabetes development, taking the Lower FA Score group as reference, and intermediate results in the other two groups. Finally, we performed a third categorization of the patients by medians of the FA Score, and we observed an increased risk of T2DM development for patients in the Higher FA Score group (unadjusted HR: 1.63; 95% CI 0.97–2.71, and adjusted HR: 1.65; 95% CI 0.94–2.89) compared with the Lower FA Score group.

**Fig. 3:**
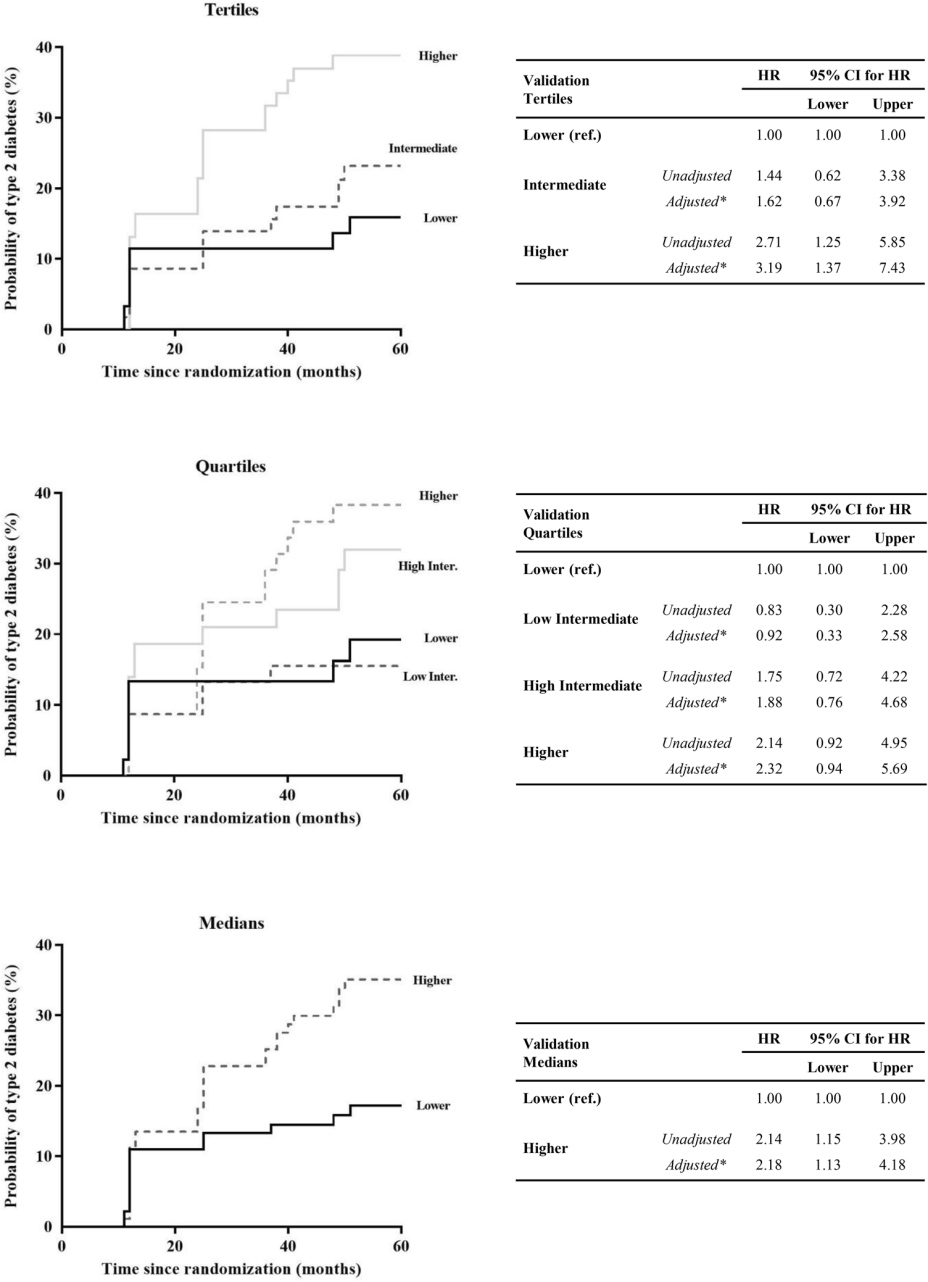
Disease-free survival by COX proportional hazards regression analysis according to FA Score in the Validation set. Patients from the Validation set were categorized according to the FA Score by tertiles, quartiles and median (ascending order). *This model was adjusted for age, gender, BMI, diet, treatment with statins, HDL-c and TG plasma levels. The hazard ratio (HR) between groups was calculated

In contrast, in the Validation set (Fig. 3), when patients were categorized by tertiles of FA Score, the Higher FA Score (unadjusted HR: 2.71; 95% CI 1.25–5.85, and adjusted HR: 3.19; 95% CI 1.37–7.43) showed a higher risk of T2DM development, taking the Lower FA Score group as reference. Similarly, regarding the second classification by quartiles, patients in the Higher FA Score (unadjusted HR: 2.14; 95% CI 0.92–4.95, and adjusted HR: 2.32; 95% CI 0.94–5.69) showed a greater T2DM risk compared with subjects in the Lower Score group, with intermediate results in the other two groups. Along the same lines, when patients were categorized by medians, we observed that patients in the Higher FA Score group had a higher risk (unadjusted HR: 2.14; 95% CI 1.15–3.98, and adjusted HR: 2.18; 95% CI 1.13–4.18) compared to the Lower FA Score group.

### Relationship between FA profile and insulin resistance and beta-cell functionality indexes

**Fig. 4:**
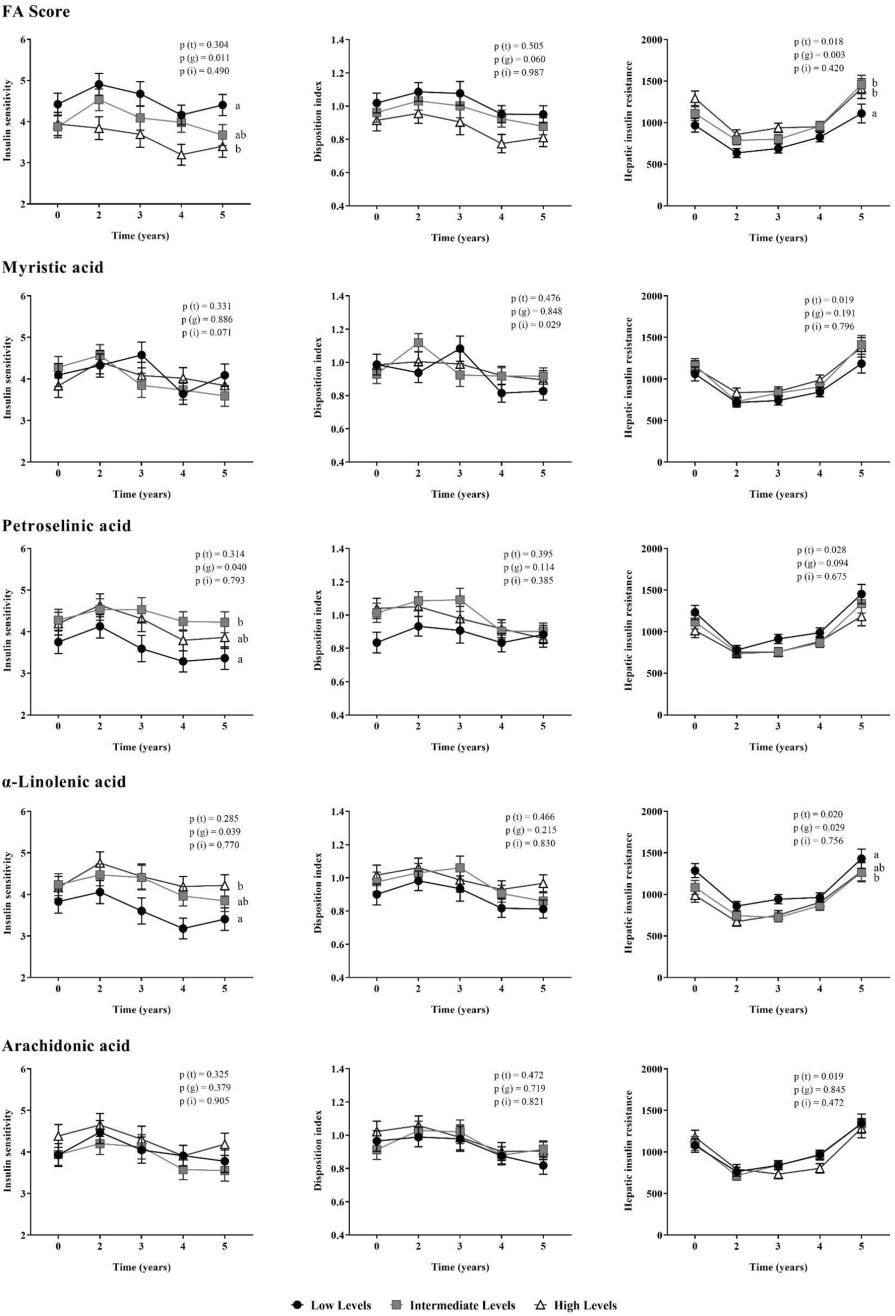
Relationship between FA profile and insulin resistance and beta-cell functionality indexes. Patients were categorized by baseline concentration of MA, PA, ALA and AA, and by FA Score calculated (ascending order). Mean ± S.E.M. of the insulin-sensitive index (ISI), disposition index (DI) and hepatic insulin resistance index (HIRI) during the follow-up period. ANOVA for repeated measures p-values adjusted by age, gender, BMI, diet, HDL-c and TG plasma levels. Global *p*-values: *P*(*t*): time effect; *P*(*g*): group effect; *P*(*i*): time by group interaction. Different letters indicate significant differences (*p* < 0.05) between groups in the Post-hoc Bonferroni's multiple comparison tests

We also evaluated insulin resistance and beta-cell function as assessed by validated indexes during 5 years of follow-up in the patients, which were categorized by ascending tertiles of the FA Score, which represent the FA profile associated to T2DM, and also by plasma levels of FA selected in the RSF (Fig. 4). When we classified our population according to the FA Score, we observed that patients included in the Low FA Score group presented higher ISI levels than patients in the High FA Score group (*p* = 0.009), and lower values of HIRI than patients classified in the Intermediate and High FA Score groups (*p* = 0.036 and *p* = 0.003, respectively), during the follow-up period.

Regarding the results of the patients classified by levels of PA, we observed that patients with low levels of PA had lower values of ISI than patients with intermediate levels of this FA (*p* = 0.038). When we categorized patients according to the ALA we found that patients with a lower ALA concentration presented lower ISI and higher HIRI values than patients who had a higher concentration (*p* = 0.044 and *p* = 0.047, respectively), during the follow-up period.

## Discussion

In our study, we identified a fatty acids (FA) profile in patients with CHD to assess the T2DM development risk by Random Survival Forest, a decision tree for prediction which sorted the different fatty acids by their importance according to the time of the T2DM development, and reduced the number of variables to 4 fatty acids from a total of 24 FA identified in plasma. Further testing in a Validation set of patients yielded reproducible results. Moreover, patients with a deleterious FA profile (higher FA Score values) have lower insulin sensitivity as determined by ISI index, and higher hepatic insulin resistance, as determined by the HIRI index.

Several studies have shown the relationship between the individual abundance of plasma FA and T2DM [13, 14]. In addition, an altered FA composition has also been related with insulin resistance [27]. Moreover, in terms of specific FA, it has been proposed that the different polyunsaturated fatty acids (PUFA), both those from plant- and marine origins, play a potentially beneficial role, although there is also a good deal of controversy. For example, the beneficial role of n3 PUFAs has been postulated for the prevention of metabolic syndrome or T2DM, but there is no conclusive evidence, and some studies have shown a lack of significant effect of n3 PUFA on glycemic control or insulin sensitivity [28, 29]. In our study, we found an inverse association between the plant-based essential n3 PUFA α-linolenic acid (ALA), the precursor of all n3 fatty acids [30], and the risk of T2DM development, whereas marine-based n3 PUFAs, such as eicosapentaenoic acid (EPA) or docosahexaenoic acid (DHA) were not selected in the model. However, some studies support the beneficial role of fish oil supplementation in T2DM prevention [31]. Our findings in the coronary heart disease population from the CORDIOPREV study are in line with previously reported findings from the study of EPIC-InterAct in a general population, which showed that ALA has a strong inverse association with T2DM risk, and that there is no association between the marine origin n3 PUFAs, EPA and DHA, and T2DM development [32]. Moreover, the relationship between plasma ALA levels and T2DM is supported by both animal and in vitro studies, which have demonstrated the capacity of ALA to regulate glucose homeostasis by affecting insulin sensitivity in different ways, such as potential functions in gene regulation, or fat metabolism [33–36]. However, human studies monitoring ALA intake have also yielded controversial results: whereas some studies have reported moderately improved fasting plasma glucose and markers of insulin resistance [37, 38], others have not shown this effect [28, 39, 40], probably because plasma levels reflect the real intake of FA more accurately than the nutritional surveys used in previous studies monitoring ALA intake. In this context, our study, in which we determined the full plasma FA composition, plasma ALA accurately assessed the T2DM risk and was also associated with insulin action. In fact, patients with higher levels of ALA showed better insulin sensitivity, with a lower insulin resistance and lower hepatic insulin resistance.

In addition to the ALA, an even-chain saturated FA, myristic acid (MA), an n6 PUFA, arachidonic acid (AA), and petroselinic acid (PA) were jointly selected by random survival forest in the model with the lowest error prediction to assess T2DM development.

In line with this, the mechanism linking T2DM and the n6 PUFA has not yet been clarified, and their proposed beneficial/deleterious effects have not been definitively proven and may depend on a complex interplay of metabolic factors [41]. In this context, γ-linolenic acid (GLA) has been described as a possible adjuvant in the prevention of T2DM [42], while other works have shown that elevated levels the GLA and its elongation product, the dihomo-γ-linolenic (DGLA), are associated with higher T2DM incidence [32]. However, in our study with coronary heart disease patients, neither of these two n6 PUFA showed a significant association with T2DM development. The most representative n6 PUFA in plasma phospholipids, arachidonic acid (AA), can be derived through the exogenous intake, since various foods are a source of AA for humans, such as lean meat and meat fats or eggs, or from linoleic acid (LA), an essential n6 fatty acid mainly found in vegetable oils. Previous works that have studied the relationship between levels of AA and T2DM yielded contradictory results [16, 43]. Here, our study showed that individually, as well as being a contributor to the FA profile identified, AA plasma levels contribute towards increasing the T2DM risk. This deleterious effect may be due to the fact that AA can be converted during the inflammatory cascade to pro-inflammatory eicosanoids, such as prostaglandins, thromboxanes or leukotrienes, which in large quantities contribute to inflammatory disorders and the formation of atheromas [44, 45] and other inflammation-related disorders such as T2DM, as the activation of inflammatory pathways has been causally linked to insulin-resistance [46]. In addition, the elevated levels of the rare and uncommon petroselinic acid (PA) in low-risk patients, which can, in turn, inhibit AA synthesis [47], suggests an overall protective role of PA against inflammation, and a preventive role for T2DM development.

Regarding MA, this FA can be derived from both exogenous intake, since it is widely distributed among plant and animal fats like butter or palm oil [48], and endogenous synthesis, being a minor product of de novo lipogenesis [49]. Our results are in line with previous works which have already shown that higher MA levels are positively associated with incident type 2 diabetes [13, 14, 50]. Moreover, the relationship between MA and diabetes risk seems to be independent of the patients’ insulin sensitivity or insulin release [50], which is in line with our results, as we did not find differences between levels of MA and insulin resistance or beta-cell functionality indexes.

Overall, the effect of plasma FA composition on T2DM development remains disputed and its roles are not still completely understood, which is partial because, most of the time, FA contribution is assessed individually or by subclasses, such as n6 or n3 fatty acids [32, 51]. To test the possibility of defining a fatty acid profile associated with T2DM development, we built a FA Score based on the contribution of several FA together, selected by RSF, and obtained an accurate prediction, which also increased after adjustment with co-variables. In fact, due to the complexity of the current diets, the FA are not consumed in an isolated way, but in combination with other FA through the nutrients, which is why we built an FA Score based on the selected plasma FA of the whole profile, and not by subclasses or individual fatty acids, thus improving the risk assessment of the FA individually. Furthermore, patients who had a deleterious FA profile (higher FA Score values) presented impaired insulin sensitivity, with higher insulin resistance and higher hepatic insulin resistance, which showed a relationship between an altered fatty acid profile and insulin sensitivity [27].

Our results also indicate that the identification of a defined fatty acid profile associated with T2DM development improves the prediction of the risk of its development as compared with the Finnish Diabetes Risk Score (FINDRISC) [52], a diabetes risk score based on anthropometric measures, blood pressure, physical activity and dietary habits, as can be seen in a previous work in which we assessed the T2DM risk to this same population [53]. Although the predictive power of this model was relatively high, the fact that several parameters are not specific for T2DM and/or are self-reported, diminishes the reliability and specificity for the prediction of T2DM status. In fact, our work highlights the need to identify novel biomarkers for the early detection of T2DM risk and/or better prognosis of T2D and to assess the efficacy of targeted interventions.

Certain limitations of the current study must be mentioned. One limitation lies in the fact that this research is based on a long-term, well-controlled dietary intervention, which ensures the quality of the study but may not reflect the level of compliance in a free-living population. Another limitation lies in the fact that the prevention of T2DM was not the primary endpoint of the CORDIOPREV trial but was rather a secondary analysis conducted in the subgroup of cardiovascular patients without T2DM at baseline. In fact, the study included a large number of patients with acute myocardial infarction, which limits our findings to people with these characteristics and precludes its generalization to healthy individuals.

In conclusion, our results suggest that an altered fatty acid profile precedes T2DM development and may be used as a predictive biomarker for the detection of patients at risk of T2DM development. In addition, our results also support the association between plasma lipid quantity and quality and insulin sensitivity, based on the relationship between a defined fatty acid profile, combined as a FA Score, and individual FA plasma levels with insulin sensitivity assessing indexes. Further studies are needed to assess the effectiveness of nutritional intervention in changing the FA profile and, therefore, preventing the development of type 2 diabetes mellitus.

## Supplementary Information

Below is the link to the electronic supplementary material.

Supplementary Fig. 1 Disease-free survival by COX proportional hazards regression analysis according to α-linolenic acid (ALA) and arachidonic acid (AA) levels. Patients were categorized by baseline concentration of ALA and AA in the Training and the Validation Set, by tertiles (ascending order). *This model was adjusted for age, gender, BMI, diet, treatment with statins, HDL-c and TG plasma levels. The hazard ratio (HR) between groups was calculated (PDF 284 KB)

Supplementary file2 (DOCX 19 KB)

Supplementary file3 (DOCX 23 KB)

## Data Availability

There are restrictions on the availability of data for the CORDIOPREV study, due to confidentiality policies. The CORDIOPREV study is an ongoing study and, therefore, databases are currently not available. After the completion of CORDIOPREV study, the datasets used and/or analysed during the current study will be available from the corresponding author on request, within reason.

## Abbreviations

ALA: α-Linolenic acid
ADA: American Diabetes Association
AA: Arachidonic acid
BMI: Body mass index
CVD: Cardiovascular disease
CORDIOPREV: Coronary Diet Intervention with Olive Oil and Cardiovascular Prevention study
CHD: Coronary heart disease
DGLA: Dihomo-γ-linolenic
DI: Disposition index
DHA: Docosahexaenoic acid
EPA: Eicosapentaenoic acid
FA: Fatty acids
GLA: γ-Linolenic acid
GC–MS: Gas chromatography-mass spectrometry
HR: Hazard ratio
HIRI: Hepatic insulin resistance index
HOMA-IR: Homeostatic model assessment insulin resistance
ISI: Insulin sensitivity index
IGI: Insulinogenic index
Incident-DIAB: Non-diabetic patients at baseline who developed T2DM after a median of 60 months of follow-up
LA: Linoleic acid
LF: Low-fat diet
MED: Mediterranean diet
MA: Myristic acid
MUFA: Monounsaturated fatty acids
Non-DIAB: Non-diabetic patients at baseline who did not develop T2DM after the follow-up period
OGTT: Oral glucose tolerance test
PA: Petroselinic acid
PUFA: Polyunsaturated fatty acids
RSF: Random survival forest
SD: Standard deviation
TAG: Triacylglycerol
T2DM: Type 2 diabetes mellitus
