# Supplementary material for: A plasma fatty acid profile associated to type 2 diabetes development: from the CORDIOPREV study
Source: Eur J Nutr. 2021 Oct 5;61(2):843–57. doi: 10.1007/s00394-021-02676-z (PMC8854256; doi:10.1007/s00394-021-02676-z)
Supplement: Supplementary file 1 — Supplementary Fig. 1 Disease-free survival by COX proportional hazards regression analysis according to α-linolenic acid (ALA) and arachidonic acid (AA) levels. Patients were categorized by baseline concentration of ALA and AA in the Training and the Validation Set, by tertiles (ascending order). *This model was adjusted for age, gender, BMI, diet, treatment with statins, HDL-c and TG plasma levels. The hazard ratio (HR) between groups was calculated (PDF 284 KB) [file 394_2021_2676_MOESM1_ESM.pdf]

European Journal of Nutrition

A plasma fatty acid profile associated to type 2 diabetes development: from the CORDIOPREV study.

Alejandro Villasanta-Gonzalez, Juan Francisco Alcalá-Díaz, Cristina Vals-Delgado, Antonio Pablo Arenas, Magdalena P. Cardelo, Juan Luis Romero-Cabrera, Fernando Rodriguez-Cantalejo, Javier Delgado-Lista, Maria M. Malagon, Pablo Perez-Martinez, Matthias B. Schulze, Antonio Camargo*, Jose Lopez-Miranda*.

[1]Lipids and Atherosclerosis Unit, GC9 Nutrigenomics, Maimonides Biomedical Research Institute of Cordoba (IMIBIC), Reina Sofia University Hospital, University of Cordoba, Spain.

[2]CIBER Fisiopatología de la Obesidad y Nutrición (CIBEROBN), Instituto de Salud Carlos III, Cordoba, Spain.

[3]Department of Cell Biology, Physiology and Immunology, University of Cordoba, Córdoba, Spain.

[4]German Center for Diabetes Research, München-Neuherberg, Germany.

[5]Department of Molecular Epidemiology, German Institute of Human Nutrition Potsdam-Rehbrücke, Nuthetal, Germany mschulze@dife.de.

[6]Germany Institute of Nutrition Science, University of Potsdam, Nuthetal, Germany.

*These authors contributed equally to this work.

Corresponding author: Prof. Jose López-Miranda and Antonio Camargo, Lipids and Atherosclerosis Unit. Reina Sofia University Hospital. University of Cordoba. Av. Menendez Pidal, s/n. 14004 Córdoba, Spain. Phone: 34-957012830. FAX: +34-957218250 email: jlopezmir@uco.es and antonio.camargo@imibic.org.

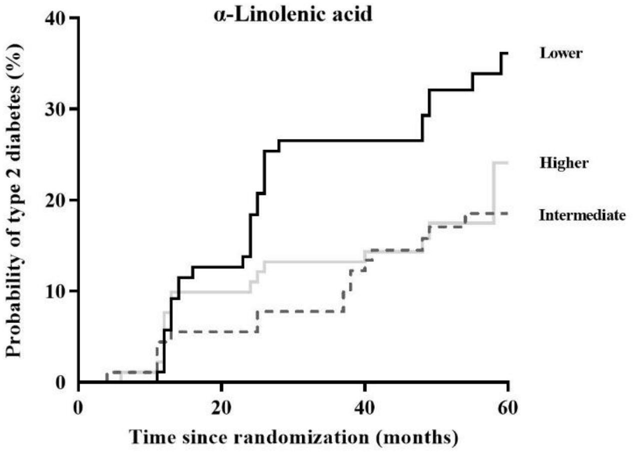

Training		HR	95% CI for HR	
			Lower	Upper
**Lower (ref.)**		1.00	1.00	1.00
**Intermediate**	*Unadjusted*	0.48	0.26	0.88
	*Adjusted**	0.42	0.22	0.82
**Higher**	*Unadjusted*	0.55	0.30	1.00
	*Adjusted**	0.56	0.30	1.07

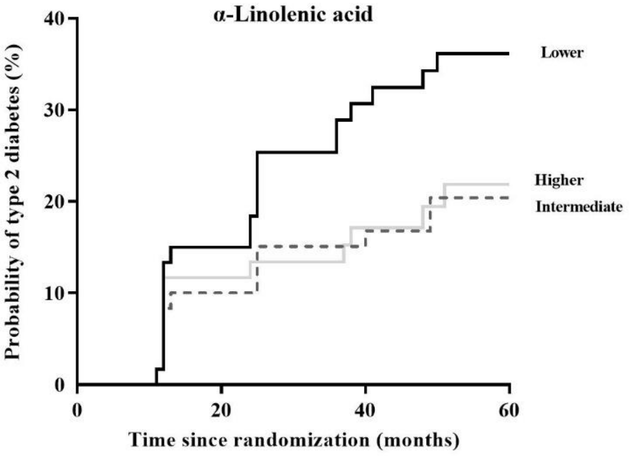

Validation		HR	95% CI for HR	
			Lower	Upper
**Lower (ref.)**		1.00	1.00	1.00
**Intermediate**	*Unadjusted*	0.52	0.26	1.06
	*Adjusted**	0.62	0.30	1.31
**Higher**	*Unadjusted*	0.56	0.28	1.14
	*Adjusted**	0.61	0.29	1.29

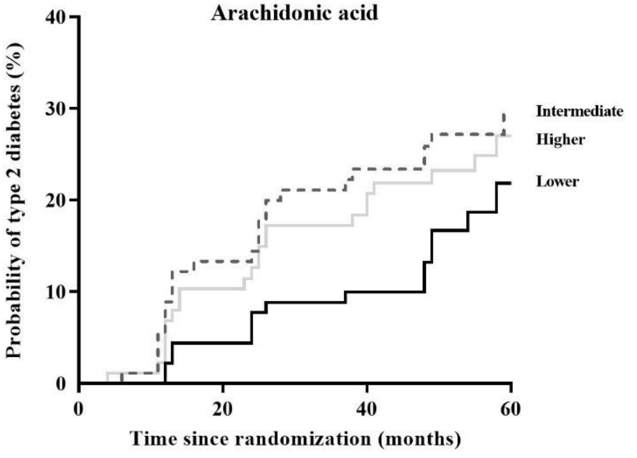

Training		HR	95% CI for HR	
			Lower	Upper
**Lower (ref.)**		1.00	1.00	1.00
**Intermediate**	*Unadjusted*	1.72	0.91	3.27
	*Adjusted**	1.91	0.98	3.74
**Higher**	*Unadjusted*	1.52	0.77	2.92
	*Adjusted**	1.74	0.85	3.55

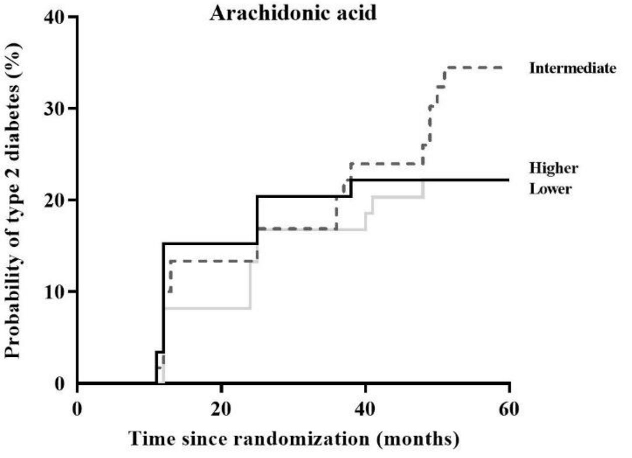

Validation		HR	95% CI for HR	
			Lower	Upper
**Lower (ref.)**		1.00	1.00	1.00
**Intermediate**	*Unadjusted*	1.46	0.72	2.96
	*Adjusted**	1.46	0.69	3.08
**Higher**	*Unadjusted*	0.96	0.44	2.06
	*Adjusted**	1.02	0.44	2.34
