# Supplementary material for: A plasma fatty acid profile associated to type 2 diabetes development: from the CORDIOPREV study
Source: Eur J Nutr. 2021 Oct 5;61(2):843–57. doi: 10.1007/s00394-021-02676-z (PMC8854256; doi:10.1007/s00394-021-02676-z)
Supplement: Supplementary file 2 — Supplementary file2 (DOCX 19 KB) [file 394_2021_2676_MOESM2_ESM.docx]

**European Journal of Nutrition**

**A plasma fatty acid profile associated to type 2 diabetes development: from the CORDIOPREV study.**

Alejandro Villasanta-Gonzalez, Juan Francisco Alcalá-Díaz, Cristina Vals-Delgado, Antonio Pablo Arenas, Magdalena P. Cardelo, Juan Luis Romero-Cabrera, Fernando Rodriguez-Cantalejo, Javier Delgado-Lista, Maria M. Malagon, Pablo Perez-Martinez, Matthias B. Schulze, Antonio Camargo*, Jose Lopez-Miranda*.

^1^Lipids and Atherosclerosis Unit, GC9 Nutrigenomics, Maimonides Biomedical Research Institute of Cordoba (IMIBIC), Reina Sofia University Hospital, University of Cordoba, Spain.

^2^CIBER Fisiopatología de la Obesidad y Nutrición (CIBEROBN), Instituto de

Salud Carlos III, Cordoba, Spain.

^3^Department of Cell Biology, Physiology and Immunology, University of Cordoba, Córdoba, Spain.

^4^German Center for Diabetes Research, München-Neuherberg, Germany.

^5^Department of Molecular Epidemiology, German Institute of Human Nutrition Potsdam-Rehbrücke, Nuthetal, Germany mschulze@dife.de.

^6^Germany Institute of Nutrition Science, University of Potsdam, Nuthetal, Germany.

*These authors contributed equally to this work.

**Corresponding author:** Prof. Jose López-Miranda and Antonio Camargo, Lipids and Atherosclerosis Unit. Reina Sofia University Hospital. University of Cordoba. Av. Menendez Pidal, s/n. 14004 Córdoba, Spain. Phone: 34-957012830. FAX: +34-957218250 email: [jlopezmir@uco.es](mailto:jlopezmir@uco.es) and [antonio.camargo@imibic.org](mailto:antonio.camargo@imibic.org).

|  | Incident-DIAB | Non-DIAB | P-value |
| --- | --- | --- | --- |
| *n* | 107 | 355 | n/a |
| *Men/Women (n)* | 87/20 | 302/53 | n/a |
| *Age (years)* | 58.75±0.87 | 57.33±0.50 | 0.171 |
| *Weight (kg)* | 85.70±1.47 | 82.49±0.72 | 0.037 |
| *BMI (kg/m^2^)* | 31.39±0.47 | 29.88±0.22 | 0.002 |
| *Waist circumference (cm)* | 105.28±1.08 | 101.73±0.57 | 0.003 |
| *Serum triacylglycerols (mg/dL)* | 132.60±6.60 | 119.45±3.24 | 0.059 |
| *Total cholesterol (mg/dL)* | 164.97±3.41 | 160.65±1.62 | 0.217 |
| *HDL-cholesterol (mg/dL)* | 43.52±1.04 | 44.58±0.53 | 0.355 |
| *LDL-cholesterol (mg/dL)* | 93.40±2.66 | 91.10±1.33 | 0.421 |
| *CRP (mg/L)* | 2.88±0.29 | 2.51±0.17 | 0.329 |
| *HbA1c ( %)* | 6.03±0.03 | 5.86±0.02 | <0.001 |
| *HbA1c (mmol/mol)* | 42.37±0.36 | 40.51±0.19 | <0.001 |
| *Fasting Glucose (mg/dL)* | 96.18±1.04 | 92.59±0.53 | 0.002 |
| *Fasting Insulin (mU/L)* | 10.51±0.66 | 8.34±0.31 | 0.001 |
| *ISI* | 3.35±0.20 | 4.32±0.14 | 0.001 |
| *HOMA-IR* | 3.37±0.30 | 2.58±0.09 | 0.001 |
| *IGI* | 0.64±0.30 | 1.08±0.06 | 0.025 |
| *Disposition Index* | 0.83±0.05 | 1.03±0.03 | 0.003 |

**Supplementary Table 1. Baseline characteristics of the population for type 2 diabetes mellitus incidence study.** Means values ± S.E.M. Incident-DIAB: patients who developed T2DM but were non-diabetic at baseline. Non-DIAB: non-diabetic patients. BMI: body mass index. HbA1c: glycated hemoglobin A1c. ISI: insulin sensitivity index. IGI: insulinogenic index. One-way ANOVA P-values.
