# Supplementary material for: A plasma fatty acid profile associated to type 2 diabetes development: from the CORDIOPREV study
Source: Eur J Nutr. 2021 Oct 5;61(2):843–57. doi: 10.1007/s00394-021-02676-z (PMC8854256; doi:10.1007/s00394-021-02676-z)
Supplement: Supplementary file 3 — Supplementary file3 (DOCX 23 KB) [file 394_2021_2676_MOESM3_ESM.docx]

**European Journal of Nutrition**

**A plasma fatty acid profile associated to type 2 diabetes development: from the CORDIOPREV study.**

Alejandro Villasanta-Gonzalez, Juan Francisco Alcalá-Díaz, Cristina Vals-Delgado, Antonio Pablo Arenas, Magdalena P. Cardelo, Juan Luis Romero-Cabrera, Fernando Rodriguez-Cantalejo, Javier Delgado-Lista, Maria M. Malagon, Pablo Perez-Martinez, Matthias B. Schulze, Antonio Camargo*, Jose Lopez-Miranda*.

^1^Lipids and Atherosclerosis Unit, GC9 Nutrigenomics, Maimonides Biomedical Research Institute of Cordoba (IMIBIC), Reina Sofia University Hospital, University of Cordoba, Spain.

^2^CIBER Fisiopatología de la Obesidad y Nutrición (CIBEROBN), Instituto de

Salud Carlos III, Cordoba, Spain.

^3^Department of Cell Biology, Physiology and Immunology, University of Cordoba, Córdoba, Spain.

^4^German Center for Diabetes Research, München-Neuherberg, Germany.

^5^Department of Molecular Epidemiology, German Institute of Human Nutrition Potsdam-Rehbrücke, Nuthetal, Germany mschulze@dife.de.

^6^Germany Institute of Nutrition Science, University of Potsdam, Nuthetal, Germany.

*These authors contributed equally to this work.

**Corresponding author:** Prof. Jose López-Miranda and Antonio Camargo, Lipids and Atherosclerosis Unit. Reina Sofia University Hospital. University of Cordoba. Av. Menendez Pidal, s/n. 14004 Córdoba, Spain. Phone: 34-957012830. FAX: +34-957218250 email: [jlopezmir@uco.es](mailto:jlopezmir@uco.es) and [antonio.camargo@imibic.org](mailto:antonio.camargo@imibic.org).

**Supplementary Table 2.** Association between fatty acids levels in an individual way and T2DM development, per SD increase. Model 1 was unadjusted and Model 2 was adjusted by age, gender, diet, BMI, treatment with statins, HDL-c and TAG plasma levels.

|  | **Coeff** | **HR** | | | **95% CI for HR** | | | **Linear trend** | | |
| --- | --- | --- | --- | --- | --- | --- | --- | --- | --- | --- |
|  |  |  |  |  | **Lower** | **Upper** | |  |  |  |
| **Model 1.** | | | | | | | | | |  |
| Myristic acid C14:0 | -0.037 | | 0.964 | 0.749 | | | 1.239 | | 0.774 |  |
| Pentadecanoic acid C15:0 | -0.080 | | 0.923 | 0.735 | | | 1.159 | | 0.490 |  |
| Palmitic acid C16:0 | 0.067 | | 1.069 | 0.819 | | | 1.397 | | 0.623 |  |
| Palmitelaidic acid C16:1t | -0.040 | | 0.961 | 0.744 | | | 1.241 | | 0.762 |  |
| Palmitoleic acid C16:1c | -0.073 | | 0.930 | 0.721 | | | 1.200 | | 0.576 |  |
| Margaric acid C17:0 | -0.131 | | 0.877 | 0.692 | | | 1.113 | | 0.280 |  |
| Stearic acid C18:0 | 0.830 | | 2.293 | 0.873 | | | 6.020 | | 0.092 º |  |
| Oleic acid C18:1c9 | -0.146 | | 0.865 | 0.704 | | | 1.061 | | 0.164 |  |
| Petroselinic acid C18:1n-12 | -0.063 | | 0.939 | 0.775 | | | 1.138 | | 0.520 |  |
| Linoleic acid C18:2n-6c | 0.026 | | 1.026 | 0.798 | | | 1.318 | | 0.842 |  |
| Arachidic acid C20:0 | -0.025 | | 0.975 | 0.754 | | | 1.261 | | 0.847 |  |
| ɣ-Linolenic acid C18:3n-6 | -0.102 | | 0.903 | 0.711 | | | 1.146 | | 0.401 |  |
| Eicosenoic acid C20:1 | -0.129 | | 0.879 | 0.678 | | | 1.139 | | 0.330 |  |
| α-Linolenic acid C18:3n-3 | -0.299 | | 0.742 | 0.571 | | | 0.964 | | 0.025 * |  |
| Eicosadienoic acid C20:2n-6 | -0.030 | | 0.970 | 0.759 | | | 1.240 | | 0.808 |  |
| Mead acid C20:3n-9 | 0.092 | | 1.096 | 0.851 | | | 1.413 | | 0.478 |  |
| Dihomo-γ-linolenic acid C20:3n-6 | 0.169 | | 1.184 | 0.911 | | | 1.539 | | 0.207 |  |
| Eicosatrienoic acid C20:3n-3 | 0.099 | | 1.105 | 0.854 | | | 1.429 | | 0.449 |  |
| Arachidonic acid C20:4n-6 | 0.211 | | 1.234 | 0.948 | | | 1.608 | | 0.119 |  |
| Eicosapentaenoic acid C20:5n-3 | 0.103 | | 1.108 | 0.871 | | | 1.409 | | 0.404 |  |
| Adrenic acid C22:4n-6 | 0.157 | | 1.170 | 0.908 | | | 1.509 | | 0.225 |  |
| Osbond acid C22:5n-6 | 0.131 | | 1.140 | 0.886 | | | 1.466 | | 0.308 |  |
| Docosapentaenoic acid C22:5n-3 | 0.147 | | 1.158 | 0.905 | | | 1.482 | | 0.244 |  |
| Docosahexaenoic acid C22:6n-3 | 0.170 | | 1.185 | 0.921 | | | 1.525 | | 0.188 |  |

|  | **Coeff** | **HR** | | | **95% CI for HR** | | | **Linear trend** | | |
| --- | --- | --- | --- | --- | --- | --- | --- | --- | --- | --- |
|  |  |  |  |  | **Lower** | **Upper** | |  |  |  |
| **Model 2.** | | | | | | | | | |  |
| Myristic acid C14:0 | -0.115 | | 0.891 | 0.664 | | | 1.197 | | 0.445 |  |
| Pentadecanoic acid C15:0 | -0.063 | | 0.937 | 0.744 | | | 1.185 | | 0.594 |  |
| Palmitic acid C16:0 | -0.027 | | 0.973 | 0.721 | | | 1.312 | | 0.858 |  |
| Palmitelaidic acid C16:1t | -0.072 | | 0.931 | 0.698 | | | 1.242 | | 0.626 |  |
| Palmitoleic acid C16:1c | -0.073 | | 0.926 | 0.696 | | | 1.233 | | 0.599 |  |
| Margaric acid C17:0 | -0.094 | | 0.911 | 0.702 | | | 1.181 | | 0.479 |  |
| Stearic acid C18:0 | 0.776 | | 2.173 | 0.725 | | | 6.512 | | 0.166 |  |
| Oleic acid C18:1c9 | -0.203 | | 0.816 | 0.640 | | | 1.042 | | 0.103 |  |
| Petroselinic acid C18:1n-12 | -0.021 | | 0.979 | 0.744 | | | 1.289 | | 0.881 |  |
| Linoleic acid C18:2n6c | 0.100 | | 1.105 | 0.836 | | | 1.462 | | 0.483 |  |
| Arachidic acid C20:0 | -0.014 | | 0.986 | 0.749 | | | 1.298 | | 0.918 |  |
| ɣ-Linolenic acid C18:3n-6 | -0.034 | | 0.966 | 0.736 | | | 1.269 | | 0.806 |  |
| Eicosenoic acid C20:1 | -0.163 | | 0.850 | 0.628 | | | 1.150 | | 0.292 |  |
| α-Linolenic acid C18:3n-3 | -0.319 | | 0.727 | 0.546 | | | 0.968 | | 0.029 * |  |
| Eicosadienoic acid C20:2n-6 | 0.094 | | 1.098 | 0.820 | | | 1.471 | | 0.530 |  |
| Mead acid C20:3n-9 | 0.136 | | 1.146 | 0.871 | | | 1.507 | | 0.331 |  |
| Dihomo-γ-linolenic acid C20:3n-6 | 0.237 | | 1.267 | 0.953 | | | 1.685 | | 0.104 |  |
| Eicosatrienoic acid C20:3n-3 | 0.141 | | 1.152 | 0.880 | | | 1.508 | | 0.303 |  |
| Arachidonic acid C20:4n-6 | 0.303 | | 1.354 | 1.015 | | | 1.806 | | 0.039 * |  |
| Eicosapentaenoic acid C20:5n-3 | 0.032 | | 1.032 | 0.790 | | | 1.348 | | 0.817 |  |
| Adrenic acid C22:4n-6 | 0.276 | | 1.317 | 0.990 | | | 1.753 | | 0.058 º |  |
| Osbond acid C22:5n-6 | 0.210 | | 1.234 | 0.941 | | | 1.619 | | 0.129 |  |
| Docosapentaenoic acid C22:5n-3 | 0.152 | | 1.165 | 0.898 | | | 1.510 | | 0.250 |  |
| Docosahexaenoic acid C22:6n-3 | 0.135 | | 1.145 | 0.873 | | | 1.501 | | 0.327 |  |
